# Interpersonal curiosity as a tool to foster safe relational spaces: a narrative literature review

**DOI:** 10.3389/fpsyg.2024.1379330

**Published:** 2024-08-13

**Authors:** Mélanie Letendre Jauniaux, Heather L. Lawford

**Affiliations:** Department of Psychology, Bishop's University, Sherbrooke, QC, Canada

**Keywords:** interpersonal curiosity, relational spaces, safer spaces, curiosity, narrative literature review

## Abstract

Interpersonal curiosity (IPC), or the desire for information about others, is a core component of human connection, belonging, security, survival, and flourishing. Current research on leveraging IPC is scarce, making it an overlooked mechanism for building safer relational spaces. This narrative literature review attempts to answer the following questions: how can IPC facilitate safe relational spaces? How can this knowledge be made accessible and actionable for readers working in relational fields or public health? Results from the analysis of 23 articles indicate that IPC can manifest as either a trait or a state. At best, IPC can be a powerful tool for connection. At worst, IPC can lead to non-prosocial behaviors and relational disruptions. Suggestions are provided to harness the potential of IPC to foster quality connection and safer relational spaces.

## 1 Introduction

This article was written for readers from various disciplines, including education, psychology, medicine, marketing, and crisis management. Through a narrative literature review, the authors aim to provide the reader with a broad and accessible overview of interpersonal curiosity (IPC) as a concept emerging from research in multiple fields conceptualized through a variety of other terms such as social curiosity, curiosity about people, and curiosity in general.

The objectives of this narrative literature review include presenting the strengths, limitations and nuances of IPC with the hope of inspiring action on multiple levels. Despite the dearth of literature on leveraging IPC to build safer relational spaces, the findings of this narrative literature review point to IPC's role as an overlooked mechanism in this respect.

This review therefore elucidates how IPC can be used to shift attitudes and behaviors toward more informed practices that facilitate safer relational spaces. Readers will acquire valuable knowledge that can be put into practice to nurture their personal and professional relationships and to navigate power structures within a workplace hierarchy.

### 1.1 Types of curiosity

The general concept of curiosity is a complex and multidimensional mechanism described by researchers as a powerful “drive to know” (Berlyne, 1954) and desire to fill a gap in knowledge (Loewenstein, 1994) about our environment, ourselves, or others. Curiosity is understood to manifest either as a positive aroused “interest” or negative sense of “deprivation” (Litman and Jimerson, 2004). An individual's cognitive assessment of their own resources to navigate this gap in knowledge will trigger certain exploratory behaviors driving human growth, motivation and active engagement (Kashdan et al., 2020). Individual differences in the expression of curiosity can be categorized in two ways. First, temporary manifestations of “states” of curiosity are elicited by the environment and the situation. Second, an individual's dispositional habits of responding with curiosity comprise the “traits” of curiosity (Kashdan et al., 2004, p. 483).

Humans seem to be driven by two distinct types of curiosity as it relates to people. When an individual's drive to know is turned inward, curiosity about oneself, or intrapersonal curiosity (InC), manifests. InC is described as an individual's interest and motivation to explore their inner world (Aschieri et al., 2018). While research has detected some overlapping neurological markers for curiosity about oneself and about others (Han et al., 2013), interpersonal curiosity (IPC), or the desire for information about other people, can be measured as a distinct construct (Litman et al., 2017; Aschieri et al., 2018; Kashdan et al., 2020). This review will focus primarily on IPC.

### 1.2 IPC and relationships

Quality relationships are fundamental to human development, learning, and thriving (Waldinger and Schulz, 2023). Connection to others and a sense of belonging are basic human needs that affect physical and mental health and ultimately survival (Baumeister and Leary, 1995; Baumeister, 2005; Tomalski and Johnson, 2010). Meeting these needs is increasingly difficult as societal levels of loneliness, anxiety, and feelings of disconnection continue to rise (Valtorta et al., 2016; Huang et al., 2021; Surkalim et al., 2022). Fortunately, IPC can help promote positive relational interactions, interesting conversations, authentic lines of inquiry, and quality connection. As a result, feelings of closeness, trust and camaraderie develop, which can serve as a strategy to de-escalate conflicts (Hartung and Renner, 2011; Kashdan et al., 2013a,b; Kolb, 2020). Furthermore, IPC could improve resilience behaviors such as tolerance of uncertainty, initiation of humor, responding nondefensively, unconventional thinking, and fostering positive social connections (Kashdan et al., 2011).

For the purpose of this article, quality relationships are defined as spaces for interaction that encompass IPC, empathy, authenticity, mindful presence, and affiliative goals (Rakel et al., 2009; Brewer et al., 2013; Kolb, 2020). Research shows that positive relationships can engender a sense of safety (Feeney and Thrush, 2010).

### 1.3 IPC and safer spaces

Relational safety refers to interactions where individuals feel secure, respected, and free from various forms of harm. Some refer to this as “brave spaces,” or “safer spaces” that encompass mutual trust, open communication, empathy, consistency and the absence of fear or threat of harm, whether physical, psychological, or emotional (SAMHSA, 2014; Ali, 2017). In a relationally safe environment, people feel comfortable expressing themselves authentically and sharing their thoughts and feelings without fear of judgment or reprisal (Shalka and Leal, 2022; Iordanou, 2023). This fosters healthier and more meaningful connections, promoting emotional wellbeing, a sense of belonging, and acceptance. Safer spaces unlock the potential for learning, connection, healing, and thriving (Perry, 2009).

## 2 Methods

This narrative literature review was conducted in five stages. First, a preliminary exploration of current relevant literature on curiosity and IPC was conducted, followed by a narrative review of best practices. Next, relevant databases, search terms, inclusion and exclusion criteria were established while building a tracking research tool in Excel. Third, articles were collected and sorted on a scale from 1 to 5 (see Appendix A). Fourth, 23 articles were selected for review and analysis (see Table 1). Lastly, the final report was written in conjunction with the development of a knowledge mobilization tool.

**Table 1:** Summary of the reviewed articles.

**References**	**Title**	**Journal**	**Key terminology of interest**	**Contributions**
Barber et al. (2021)	Social anxiety and the generation of positivity during dyadic interaction: curiosity and authenticity are the keys to success	Behavior Therapy	Curiosity	Keeping affiliative goals in mind during an interaction can promote positive outcomes by increasing curiosity and felt authenticity for both individuals within a dyad. This association is slightly more modest for those with high trait social anxiety. When interacting with another individual, keeping affiliative goals in mind can benefit relational outcomes for both parties.
Derby et al. (2012)	Snooping in romantic relationships	College Student Journal	Snooping	Snooping is motivated by covert curious exploration and seems common among the undergraduate students surveyed. This means of coping with uncertainty was shown to have potentially adverse effects on both partners as well as the relationship. When considering engaging in covert exploratory behaviors such as snooping, one should carefully weigh the benefits and the costs.
Guthrie (2009)	Be curious	Negotiation Journal	Curiosity	Curiosity is presented as the most important skill for successful negotiators. To enhance one's curiosity, the authors suggest strategies such as (1) varying tasks to find novelty, (2) increasing a task's level of complexity and challenge, and (3) focusing on the purpose of the interaction.
Han et al. (2013)	Electrophysiological evidence for the importance of interpersonal curiosity	Brain Research	Interpersonal curiosity	Information about the outcome of an interactive gambling task seems to be of higher value when the opponent was human vs. a computer. This shows interpersonal information as a significant source of social motivation in the human brain.
Hartung and Renner (2011)	Social curiosity and interpersonal perception: a judge × trait interaction	Personality and Social Psychology Bulletin	Social curiosity	High socially curious individuals seem to be more accurate judges of certain personality traits such as Extraversion and Openness. Their accuracy appears to be related to a more proficient use of social cues. More accurate judgment of traits may help highly curious individuals to navigate successful relational interactions.
Hartung et al. (2013)	Social curiosity and gossip: related but different drives of social functioning	PLoS ONE	Social curiosity	Humans need to gather and convey social information in order to navigate their complex worlds. This need may arise from two overlapping but different mechanisms, namely gossip and social curiosity. Despite both serving as critical drives to learning and relationship building, gossiping can serve entertainment purposes, whereas social curiosity is likely driven more so by a need to belong and gain insight into another's feelings and thoughts.
Huang et al. (2021)	How anxiety predicts interpersonal curiosity during the COVID-19 pandemic: the mediation effect of interpersonal distancing and autistic tendency	Personality and Individual Differences	Interpersonal curiosity	Mandatory social isolation during the COVID-19 pandemic along with anxiety positively correlated with autistic tendencies. Furthermore, distancing and autistic tendencies negatively predicted IPC. Pandemic-related social isolation might have interacted with other factors to lower curiosity about others among certain individuals.
Kashdan et al. (2004)	Curiosity and exploration: facilitating positive subjective experiences and personal growth opportunities	Journal of Personality Assessment	Curiosity	The authors present curiosity as a construct of high relevance to many fields of research. They expand on a theoretical model and the design of a new measure: the Curiosity and Exploration Inventory (CEI). The CEI shows promise, and good psychometric properties to measure curiosity more broadly.
Kashdan et al. (2011)	When curiosity breeds intimacy: taking advantage of intimacy opportunities and transforming boring conversations	Journal of Personality	Curiosity	Curiosity can contribute to and generate the feeling of closeness and subsequent overall positive encounters between strangers. The highly curious individual is more likely to generate a more enjoyable conversation for both parties. This holds true in intimate discussions as well as superficial small talk.
Kashdan et al. (2013a)	Curiosity protects against interpersonal aggression: cross-sectional, daily process, and behavioral evidence	Journal of Personality	Curiosity	Curiosity seems to be inversely related to aggression, whether it is measured as a state or trait. However, context can influence the expression of aggression. For example, some individuals are more likely to act aggressively toward partners in a close relationship than toward strangers. Despite some nuances, curiosity remains a valuable safeguard against interpersonal aggression and a trait that promotes resilience.
Kashdan et al. (2013b)	How are curious people viewed and how do they behave in social situations? From the perspectives of self, friends, parents, and unacquainted observers	Journal of Personality	Curiosity	A strong positive correlation was found between how an individual rates their own curiosity and how others (friends, parents and observers) rate that individual's curiosity. Curious individuals seem to exhibit resilience and adaptive behaviors such as tolerance of uncertainty, initiation of humor, and unconventional thinking.
Kashdan et al. (2018)	The five-dimensional curiosity scale: capturing the bandwidth of curiosity and identifying four unique subgroups of curious people	Journal of Research in Personality	Curiosity/ Social curiosity	The authors posit a multi-dimensional model of curiosity along with the five-dimensional curiosity scale. This tool aims to capture individual differences in experiencing and expressing curiosity to broaden the dualistic understanding and measurements of this trait/state. The five dimensions presented by the authors are (1) Joyous exploration, (2) deprivation sensitivity, (3) stress tolerance, (4) thrill seeking, and (5) social curiosity. Furthermore, the authors highlight links between some dimensions of curiosity, wellbeing and health. Other dimensions were found to be unrelated or even negatively related. The healthiest outcomes are observed in those with high joyous exploration, stress tolerance, thrill seeking, and low deprivation sensitivity.
Kashdan et al. (2020)	The five-dimensional curiosity scale revised (5DCR): briefer subscales while separating overt and covert social curiosity.	Personality and Individual Differences	Curiosity/Social curiosity	The authors revised the five-dimensional curiosity scale. The fifth dimension of curiosity, that of social curiosity, was modified to distinguish between overt and covert types of social curiosity. The authors removed weaker items, thereby shortening the scale.
Kashdan and Roberts (2004)	Trait and state curiosity in the genesis of intimacy: Differentiation from related constructs	Journal of Social and Clinical Psychology	Curiosity	Curious individuals seem to generate positive relational outcomes during interactions through state curiosity. This positive outcome was not dependent on their level of state positive affect. One's level of social anxiety, however, influenced the relationship between curiosity and perceived affinity with a confederate. This work points to the benefits of curiosity as a catalyst for reciprocal self-disclosure and developing closeness in novel relational contexts.
Kashdan and Roberts (2006)	Affective outcomes in superficial and intimate interactions: roles of social anxiety and curiosity	Journal of Research in Personality	Curiosity	Individual differences in trait curiosity and social anxiety can predict affect and subjective experience during an interaction. Positive affect was more closely associated with curiosity than negative affect. Negative affect was associated with social anxiety depending on social context. Curiosity can lead to more favorable exploratory behaviors, and positive interpretation of interpersonal experiences.
Kolb (2020)	Making connection as critical moves in negotiation.	Negotiation Journal	Curiosity	Critical moments are reported to shaping the trajectory of a negotiation process. These important turning points, as described by multiple experienced negotiators, seem to rely on moves toward connection. Indeed, a series of verbal and nonverbal strategies allow the negotiator to build trust and develop rapport. Experienced negotiators in crisis situations emphasize authentic interpersonal curiosity and genuine connection.
Litman and Pezzo (2007)	Dimensionality of interpersonal curiosity	Personality and Individual Differences	Interpersonal curiosity	Three main dimensions of interpersonal curiosity were identified: curiosity about emotions, spying and prying, and snooping. The authors developed a scale to measure the dimensions of interpersonal curiosity.
Renner (2006)	Curiosity about people: the development of a social curiosity measure in adults	Journal of Personality Assessment	Social curiosity	Two types of social curiosity emerged from the social curiosity scale developed by the author: general and covert social curiosity. This finding corroborates the notion that social curiosity stems from different motivations and drives and is influenced by context. Furthermore, anxiety in social interactions facilitates covert social curiosity and inhibits general social curiosity.
Robinson et al. (2017)	Adult life stage and crisis as predictors of curiosity and authenticity: testing inferences from Erikson's lifespan theory	International Journal of Behavioral Development	Interpersonal/ intrapersonal curiosity	Adults in developmental crisis exhibit heightened curiosity and decreased felt authenticity.
Shields et al. (2013)	Pain assessment: the roles of physician certainty and curiosity	Health Communication	Curiosity	Pain assessment is complicated by the lack of objective measures of pain. Furthermore, overworked physicians may employ cognitive shortcuts and communication styles, such as verbal and nonverbal certainty, that stifle curious exploration. Premature closure and incomplete assessments can result. Alternatively, physicians who express concern and curiosity seem to offer superior pain assessments.
Thomas and Vinuales (2017)	Understanding the role of social influence in piquing curiosity and influencing attitudes and behaviors in a social network environment	Psychology and Marketing	Curiosity	Social group belonging depicted in advertisements correlated with consumer curiosity levels, attitudes and behaviors. Out-group advertisement is more effective for consumers with higher susceptibility to interpersonal influence.
Watanabe et al. (2020)	Social relationships and functional status among Japanese elderly adults living in a suburban area	Public Health	Social curiosity	In this prospective cohort study, Japanese adults aged 65 and older were assessed at two time points, 6 years apart. Those who scored low in social curiosity had greater functional and cognitive decline and higher mortality rates than their more socially curious peers.
Zhang (2019)	Reliability and validity of the social curiosity scale among Chinese university students	Social Behavior and Personality	Social curiosity	The authors assessed the social curiosity scale with Chinese university students. The authors posit that the Chinese version of this scale could be used to assess differences in social curiosity among Chinese university students to offer them tailored support.

### 2.1 Search terms, databases, and search results

Since IPC is not an index term, the main Index term/SU Subject term/MeSH Major Topic used was “curiosity.” To refine the search, the key words “interpersonal curiosity” were then added. Additional filters included peer reviewed articles from 1997 to 2022, the data of which was gathered from adult populations. Six databases were explored to narrow down the relevant literature. This search yielded a total of 146 peer-reviewed articles about curiosity that mentioned interpersonal curiosity.

The principal author developed a rating scale to assess 146 papers from 1 to 5. This relevance rating scale was developed for this project based on relevance to IPC and the research questions (see Appendix A). Supporting documents were also selected from the literature to contextualize the main articles and strengthen the theoretical framework.

The first database, PsycNet, yielded 41 results, 17 of which were included in the final analysis. PubMed provided 21 results, seven of which had previously been identified through PsycNet; only one was included in the final analysis. A search of the Psychological and Behavioral Science database rendered eleven results, five of which had been identified through previous databases, and two were included in the final analysis. The Academic Source Complete database provided 33 results, 19 of which had previously been identified through other databases, and of those remaining, only one was selected. ERIC rendered two results, neither of which qualified for the final analysis. Business Source Complete yielded a total of 12 results, three of which had been previously identified; one was used for the analysis. Twenty-three of these articles scored a 5 owing to their focus on curiosity within interpersonal contexts and were used for the final analysis of the narrative literature review.

## 3 Results

### 3.1 The operationalization of curiosity and methods from the literature

The variety of empirical methods employed in the articles selected for this literature review yielded multiple markers and operationalizations of curiosity. Terminology corresponding to IPC in the 23 studies included social curiosity, curiosity about people, and curiosity in general (see Table 1). In order to encompass these related constructs, the authors utilized the emerging term “IPC” throughout the text. Though no single study is perfect, and the construct of IPC is not always easy to measure, the strength of the narrative literature review is its ability to integrate various methods, thereby reducing potential limitations.

Twenty of the 23 articles used self-reports as part of their methodology, often in combination with observed behaviors during laboratory tasks, scales, longitudinal designs, etc. Five of the articles detailed the development or validation of scales, broadening our understanding and capacity to capture more aspects of IPC (Renner, 2006; Litman and Pezzo, 2007; Ye et al., 2015; Zhang, 2019; Kashdan et al., 2020). Kashdan et al. (2020) updated their 2018 5DC scale (Kashdan et al., 2018) as a measure of IPC. This revised scale was validated through a survey of 943 adults through Amazon's Mechanical Turk and a follow up with participants after 2 months and then again after 8 months. The questionnaire assessed 5 dimensions of IPC: joyous exploration, deprivation sensitivity, stress tolerance, thrill-seeking, and both overt and covert IPC. Findings support the notion that IPC correlates with personality traits such as extraversion while being a function of different motivations and mechanisms.

IPC was also measured through brain activity. Han et al. (2013) measured IPC with EEGs during an interactive gambling task that induced a reaction in the brain to self and others, but very little response to the anticipation of feedback from a computer.

### 3.2 The anatomy (or the essence) of IPC

The concept of IPC seems to emerge as a core component of human flourishing. Indeed, human survival, belonging, and cultural transmission hinge on collecting and sharing important information about self, others, and the world. Such information is gained from reflecting on the self in relation to social and cultural environments (Baumeister, 2005). The value of social information may explain why humans can remain curious about themselves and others over the course of their lifetime and why an understanding of the underlying processes may be important for those working within relational fields.

### 3.3 Advantages of exhibiting IPC

Research underlines a number of positive outcomes associated with IPC. Research suggests that this habit not only benefits the individual, but also the quality of the interactions with those around them who detect their level of IPC (Kashdan et al., 2013b). Indeed, a person's level of IPC can be perceived by new acquaintances fairly accurately, according to Kashdan et al. (2013b). Using a multi-method design, the researchers analyzed self/parent/friend-reported data from 220 American undergraduate students. This data was combined with third party observer codings for 167 of the 220 participants whose behaviors were assessed during a 5-minute interaction task with a stranger. Results showed that IPC is visible to others and tends to facilitate bonding (Kashdan et al., 2013b).

Hartung and Renner (2011) found that when navigating their social worlds, not only can interpersonally curious individuals be easily identified, they are more skilled at detecting and using social cues to identify the personality traits of others, such as extraversion and openness. Using social information more effectively could give individuals with high IPC adaptive advantages in navigating relationships as well as in avoiding rejection and exclusion (Hartung and Renner, 2011). Indeed, Kawamoto et al. (2017) hypothesized that being less affected by social rejection may allow individuals with IPC to better adapt psychologically and to more effectively generate intimacy in social interactions. This seemed to deepen the sense of belonging and increase reciprocal satisfaction (Kashdan et al., 2011; Kawamoto et al., 2017). Ye et al. (2015) also showed that students with high levels of IPC were more satisfied with their university experience, more agreeable, open to change, and valued growth and adaptation in their social worlds. Indeed, interest in the internal experiences of others is linked to empathy, emotional intelligence, and self-compassion (Litman and Pezzo, 2007; Bluth et al., 2018; Barber et al., 2021).

IPC can bring multiple positive outcomes, including growth, learning, quality connections, and more (Kashdan and Roberts, 2004; Kashdan et al., 2013a; Barber et al., 2021). Furthermore, IPC correlates positively with good psychological flexibility, conflict resolution, and the ability to navigate ambiguities (Kashdan et al., 2013a,b). Successful individuals with high IPC who also demonstrate stronger listening skills and true empathic concern for the wellbeing of others, create meaningful relationships (Kashdan et al., 2013b). However, IPC must be contextualized within a balance of person-environment interactions and knowledge of IPC's potential for harm (Derby et al., 2012).

### 3.4 Environmental and contextual considerations

Readers are encouraged to consider how IPC levels are modulated by environmental factors such as boring, intrusive, or stressful social situations (Kashdan and Roberts, 2004; Kashdan et al., 2004). Under stressful circumstances, certain individuals will move into an exploratory behavior and others will not.

Individuals low in IPC can be more likely to cope with social stressors using covert mechanisms and aggression (Hartung and Renner, 2011; Derby et al., 2012). Indeed, Kashdan et al. (2013a) showed that IPC correlates negatively with aggression in most relationships. In certain contexts of perceived danger, exploration can be inhibited or covert exploration used, thereby hindering healthy relational outcomes (Kashdan and Roberts, 2006; Feeney and Thrush, 2010; Porter et al., 2020). For example, the stress associated with the COVID-19 pandemic caused some individuals to adopt greater autistic-type tendencies in reaction to social distancing measures. In a sample of 1,071 participants, Huang et al. (2021) explored the relationships between social distancing, IPC, and autistic tendencies during February and March of 2020. The researchers identified adherence to social distancing and anxiety levels as being positively correlated with autistic tendencies, which yielded lower levels of IPC-related exploration. Researchers noted that the relationship between anxiety and IPC remains complicated and in need of further investigation. In truly unsafe relational spaces, tempering one's desire to explore can be the most adaptive, even lifesaving, response.

Some potential motivations driving IPC were explored by Litman and Pezzo (2007) who found that the pleasure of learning about others can stem more from the discomfort of not knowing, suggesting that diminishing uncertainty about others brings satisfaction (Litman and Jimerson, 2004). Indeed, IPC can function to reduce uncertainty and establish a feeling of safety in relational spaces (Litman, 2016).

Renner (2006) developed a social curiosity scale showing how IPC can be mediated by social anxiety. Highly socially anxious individuals may be equally as curious as their low anxiety counterparts; however, the behavioral manifestations of their IPC tend to be more covert. These covert behaviors of IPC can include gossiping and snooping which are not associated with the same positive outcomes as overt IPC. Hartung et al. (2013) posited that similar to IPC, gossip can be a mechanism through which humans learn, transmit culture, and set norms. Gossip is motivated by the urge to influence others, share norms, exclude nonconformists, and entertain others (Hartung et al., 2013). Snooping is another covert IPC behavior that may lead to unintended consequences. Derby et al. (2012) explored snooping in romantic relationships through the lens of uncertainty reduction theory. The researchers observed that snooping among the sample population was frequently carried out to reduce uncertainty in relationships and establish predictability of a partner's behaviors. One example is checking a partner's phone for text conversations. Fueled by IPC and suspicion of cheating, female partners who had been betrayed and reported feeling jealous seemed to engage in more frequent snooping behaviors. The researchers caution that snooping can be associated with more negative than positive outcomes in relationships such as decreased trust, increased conflict, and worse relationship outcomes. This study shines a light on how IPC and its associated behaviors can lead to negative outcomes when a person is unable to balance their need for information with respect for another's personal privacy.

Research shows that individuals adapt their behavior depending on IPC and situational factors, one's own behaviors can also influence IPC. Indeed, adopting non-prosocial behaviors can have an impact on the expression of exploratory behaviors as demonstrated by Schmidt et al. (2020). Their study put participants in a virtual dictator game where they were randomly assigned to fair or unfair conditions and could pay for feedback about their performance or feedback about other players' reactions (both were indicators of IPC). All 117 participants seemed to demonstrate IPC; however, those who had engaged in non-prosocial behaviors under unfair conditions were significantly more likely to pay to avoid feedback rather than to get feedback about their performance, indicating less curiosity. Shame and fear seemed to be the key components behind the choice of these non-prosocial participants (Schmidt et al., 2020).

Individuals with high IPC seem to practice a healthy balance between concern for others and the self. Curiosity about people, whether directed toward the self or others, seems to overlap (Han et al., 2013; Litman et al., 2017). Aschieri et al. (2018) present InC as a function of two factors, the individual's attitude toward InC and their interest in gaining more InC. Litman et al. (2017) found that individual differences in InC emerged through three main factors: understanding one's feelings and motives, reflecting on one's past and exploring one's identity and purpose. Furthermore, the authors posited high InC correlated with the individual scoring low on reported self-knowledge, having more sensitivity to others' emotions and gestures, engaging in more private introspection, experiencing more distress, and feeling concerned about coping with threats.

InC can be a strong motivator of exploration and learning, as evidenced by Robinson et al. (2017) who investigated developmental crises in adulthood. Their research was inspired by Erikson's (1994) view of crises as generators of uncertainty that stimulate curiosity and lower the congruence of self and perceived authenticity. Using a quasi-experimental design, they compared crisis and non-crisis data from 963 participants from early, midlife, and later life phases. Participants were asked to complete self-reporting curiosity scales and crisis self-appraisal. Adults in crisis showed significantly more curiosity, especially IPC, and curiosity toward the world, but weaker feelings of authenticity. While being more curious can lead to discomfort, these gaps in perceived knowledge during developmental crises could lead to growth, even more curiosity, and learning about the self if handled with self-compassion (Bluth et al., 2018). Intrinsic factors contributing to IPC and InC can include anxiety (Renner, 2006; Barber et al., 2021), attachment style (Mikulincer, 1997), and personality (Jach and Smillie, 2021).

Marketing research shows that the need to belong stimulates IPC and can be used as a powerful tool to shape consumer attitudes and behaviors. Thomas and Vinuales (2017) studied curiosity in relation to marketing, exploring the premise that the brain is wired to react strongly to membership cues that categorize others in two ways: similar and different. Through a task and a self-reporting questionnaire, they measured the attitudes, curiosity, and behavioral intent of participants after seeing an advertisement featuring actors who were either similar to themselves or different. Results showed that participants who identified with the actors exhibited greater IPC, positive attitudes, and intent to purchase. If, however, the participant had reported higher baseline social preoccupations, they were influenced by dissimilar actors as well.

### 3.5 Fostering relational safety

Relational safety requires a certain level of intimacy, which is mediated in part by IPC according to Kashdan et al. (2020) and Obert (2016). Intimacy is described as the product of four states: curiosity, empathy, vulnerability, and recognizing irreducibility–the realization that one can never fully know the other's internal reality. Certain combinations of these states must occur for affective outcomes to be positive. For example, IPC without empathy can lead to aggression, and without irreducibility, intimacy could become utilitarian self-gratification (Obert, 2016). Likewise, vulnerability devoid of IPC could lead to selfishness.

Safe relational spaces are important in many spheres of life, such as the medical field. Shields et al. (2013) showed that relational outcomes of a doctor-patient consultation, mediated by levels of IPC, can impact pain management for patients. More specifically, a physician's attitude of certainty arising from cognitive shortcuts or subconscious stereotypes may result in less thorough consultations, thereby discouraging patients from fully expressing their concerns. This study recruited actors trained to pose as patients to make visits to 40 medical physicians, 20 of whom were specialists and 20 were family doctors. The consultations were recorded and assessed for fidelity; several weeks later, a manipulation check assessed if physicians had detected the actors. The accuracy of each physician's pain assessment was correlated with their use of certainty language and tone of voice indicators of anxiety and/or concern. Doctors who demonstrated more IPC in their exploration of the patient's story showed more complete and thorough assessments of the patient's pain with less premature closure. Those who had more behavioral markers of IPC including empathy in both their tone of voice and nonverbal cues also showed better pain assessment. This genuine IPC probably required more of the doctors' cognitive resources, but it also meant less certainty and more exploration in their interactions with patients, which led to more thorough assessments (Shields et al., 2013). Physicians could be trained to practice greater IPC because this can lead to more accurate diagnoses and better health and wellbeing outcomes (Rakel et al., 2009).

### 3.6 Fostering IPC and safety in public health

From a public health standpoint, Watanabe et al. (2020) highlight IPC as a potential protective factor against cognitive and functional decline in elderly populations. Their prospective cohort study was conducted on a sample of 674 Japanese adults over 65 years of age at one time point and again six years later. Researchers assessed changes in health and functional autonomy as well as mortality, finding that poor IPC and social interaction were tightly linked with poorer health outcomes. The data also revealed that social relationships can impact health through two main pathways: (1) buffering the impacts of stress, and (2) increasing one's sense of belonging, purpose, and motivation to adopt positive health behaviors (Watanabe et al., 2020). Therefore, implementing programs that promote connection and IPC should be a primary objective among public health officials seeking to undo some of the harms caused by global crises such as pandemic-related lockdowns and the loneliness epidemic (Valtorta et al., 2016; Magruder et al., 2017).

Institutions and workplaces that adopt trauma-informed approaches could be a promising avenue for the promotion of safer spaces and IPC through policy, practice and public health priorities (SAMHSA, 2014). The literature shows that individuals experiencing social anxiety (Barber et al., 2021), that have sensitized stress responses (Arnsten, 2015), neural developmental issues (Twardosz and Lutzker, 2010; Tottenham and Galván, 2016; Garvin and Krishnan, 2022), and attachment struggles (Kashdan et al., 2011) develop certain worldviews and cope as best they can through adversity. This coping can become maladaptive, however, when such views restrict exploration in healthy environments. Teaching individuals to engage in IPC as part of a trauma-informed approach can assist in the development of safer relational spaces (SAMHSA, 2014; Kelly, 2015; Stanley and Van der Kolk, 2019).

### 3.7 Practical tips and tricks

A greater awareness of one's own IPC habits, with consideration of context, can improve social interaction (Barber et al., 2021). This self-awareness can be accomplished through creative writing and journaling about one's barriers to IPC (Brynne et al., 2019). Readers can also learn more about IPC through infographics, books, and articles like this one.

Depending on one's profession, teaching others about the value of IPC can shift a community toward a more inclusive, connected culture (Grossnickle, 2016). The “ABCD” of IPC, summarized in Table 2, offers a succinct guide to teaching the mechanisms of IPC from stimulus to behaviors. Educators are invited to invest in quality connections first and foremost. They are also encouraged to hold the position of the one who “does not know” putting aside preconceptions and judgments in order to hold space to truly listen, build trust, and maintain healthy relationships (Gilligan and Eddy, 2021; Alessi and Kahn, 2023).

**Table 2:** The ABCD of curiousity.

**A**	**Awareness of a gap in knowledge (Loewenstein, 1994; Kashdan et al., 2004)**	**Bottom-up: reaction Top-down: intention**
**B**	Body activation elicits a response (Loewenstein, 1994; Litman, 2005; Litman and Silvia, 2006; Kashdan et al., 2020)	Discomfort of not knowing, also known as deprivation sensitivity Interest and pleasure toward acquiring new information, also known as joyous exploration
**C**	Cognitive appraisal of a gap in knowledge (Silvia, 2005; Kashdan et al., 2020)	Is there an opportunity for growth, is it novel and interesting information? Does the individual have the coping skills required to engage despite uncertainty?
**D**	Direction of behaviors (Kashdan et al., 2020)	Overt Covert

During social interactions, actively looking for similarities and identifying affiliative goals with the other are two concrete and accessible techniques for those looking to foster IPC in relational spaces (Thomas and Vinuales, 2017; Kolb, 2020; Barber et al., 2021). Furthermore, actively allowing others to preserve their sense of control and agency when tensions arise is more likely to generate receptivity, engagement, and high-quality interactions (Silvia, 2005; Kolb, 2020). Remaining in the discomfort of not knowing may feel counterintuitive at times, but doing so is necessary to maintain healthy levels of IPC.

Engaging in IPC with authentic and affiliative goals in mind also serves to sustain positive social interactions despite varying levels of social anxiety, according to Barber et al. (2021). The researchers found that individuals with both high and low social anxiety benefited from affiliative goals and engagement, which seems to enhance IPC, authenticity, and positive affect in social situations. In other words, devoting energy to affiliative goals allows one to prioritize connections over modulating one's reactions. Increased authenticity during an interaction often leads to an internalized sense of relational success whereby the positive outcome is attributed to the “real self” rather than the safety behaviors and reflexes engaged when feeling inauthentic.

When connecting with others during a time of crisis, the literature on effective negotiation highlights several key considerations (Guthrie, 2009). For example, Kolb (2020) analyzed transcripts and teaching documents from internationally renowned negotiators who actively attempt to understand the motivation of others. Establishing and maintaining a non-judgmental space that can hold the other's narratives or “stories” cultivates trust. When combined with active participation, balanced power, and mutual agency, defenses may be lowered and better outcomes may be achieved.

Furthermore, communication techniques such as valuing moments of silence, asking questions, reversing roles, and allowing the other to save face have been shown to build trust, foster a sense of safety and mutual respect, and engender critical turning points when faced with a relational impasse. Connecting through empathy and IPC, while hardly a simple task, represents an important strategy in reframing difficult interpersonal standoffs to ultimately create safer and more mutually enriching relational spaces (Kolb, 2020).

### 3.8 Working from state to trait IPC

While momentary exploration may be described as a “state” of IPC, engaging in frequent practice of IPC leads to an increased likelihood of IPC “trait” acquisition (Kashdan et al., 2011). IPC is not a fixed trait; rather, the brain's plasticity allows for the adoption of IPC habits and traits (Kashdan and Roberts, 2006). Indeed, with practice, individuals can gradually modify their undesirable responses to gaps in knowledge such as by decreasing covert IPC behaviors and deriving more satisfaction through overt IPC (Kashdan and Roberts, 2004; Ludwig et al., 2020).

IPC habits should be modified with a skillful emphasis on developing overt IPC behaviors when appropriate since measures of covert and overt IPC have been associated with different outcomes (Kashdan et al., 2020). Overt IPC was linked to positive psychological outcomes and moderated intellectual humility and wisdom. Additionally, overt IPC fostered empathy and common humanity in the face of conflict. Kashdan et al. (2018) posited that individuals with overt trait IPC are more likely to be motivated by personal growth and the welfare of others. Overt trait IPC can help individuals foster a sense of belonging and common humanity, making the intentional practice of IPC a worthy goal for those wishing to generate these positive outcomes.

Finally, modeling healthy IPC exploration can aid others in learning to apply this valuable skill. It is important to practice IPC with patience and understanding. Remembering the complex interplay of personal and situational factors can be critical to avoiding misunderstandings and fostering positive relational outcomes. Practicing exploration behaviors may pose a challenge for individuals whose lived experience and stress response have hindered their capacity for adaptive coping. Indeed, venturing into the unknown may signal danger for some, regardless of the good intentions of those around them. Modeling IPC, therefore, can help all parties reduce judgment, increase compassion, and foster connection.

## 4 Summary

This review of the literature on IPC aimed to summarize the relevant scholarly findings and investigate how IPC could be used as a tool to foster safe relational spaces. The work of experts from various disciplines was reviewed, namely that of negotiators, neural psychologists, social psychologists, cognitive psychologists, as well as experts from the fields of education, medicine, marketing, and economics. These authors contributed different pieces of a larger puzzle that speaks to the potential of IPC to benefit relational spaces. IPC must be considered within its context, complexities, and person-environment interactions.

The findings are presented in eight sections. The first section reviews the operationalization of curiosity and methods from the literature. The second section expounds on IPC as an emerging construct. The third section details the advantages of overt IPC in relational encounters. The fourth section explores elements of environmental and contextual considerations that modulate IPC and exploratory behaviors. For example, an individual's attachment, stress levels, lived experience, InC and sense of group membership can also influence the behavioral expressions of IPC.

The fifth section illustrates how fostering relational safety through intimacy and empathy allows for positive outcomes such as better conflict resolution and diagnostic accuracy. The sixth section makes the case for IPC on a broader scale such as in public health. Trauma-informed approaches incorporating IPC are mentioned as initiatives worthy of exploration. Policymakers should consider initiatives that promote IPC for the sake of greater community resilience.

The seventh section offers the reader practical tips and tools to developing their own IPC. Readers are offered strategies that can be employed during interactions such as keeping affiliative goals in mind during an exchange, actively looking for similarities between oneself and the other person, prioritizing the connection rather than outcomes, actively allowing the other to preserve a sense of control and agency, holding a non-judgmental space to truly listen to the other's story, teaching others about IPC, and most importantly modeling IPC.

The eighth section discusses IPC development from state to trait. Although IPC can manifest as a state or trait, individuals can develop trait IPC through intentional and repetitive practice (Kashdan and Roberts, 2004). Individuals who habitually engage in IPC develop a positive feedback loop whereby they are more likely to interpret novel situations as opportunities to learn, which reinforces their IPC habits and changes their brain's responses to stimuli (Kashdan et al., 2011; Brewer et al., 2013). Several benefits to developing overt trait IPC include greater psychological flexibility and healthier coping and communication.

In sum, IPC has not attracted the research interest it deserves until very recently. The hope is that this review will resonate with readers who will in turn inspire meaningful conversations and change. Much remains to be discovered in the field of IPC.

### 4.1 Future directions

Basic and applied research that delves further into IPC, especially coupled with trauma-informed approaches, are worthy endeavors. Research using longitudinal designs and brain measurements could help further our understanding of how IPC develops and impacts lives. Additionally, barriers to adopting IPC should be further documented.

The relationships between InC and IPC represent another gap in the research that could be further explored, as stipulated by Litman et al. (2017) and Aschieri et al. (2018). Such studies could inform our understanding of their complex and somewhat overlapping natures. The interplay of psychological flexibility, curiosity, and life outcomes also merits additional study.

### 4.2 Strength and limitations of narrative literature reviews

Advantages inherent to narrative literature reviews include the ability to survey diverse methodologies and to analyze the pattern of results elucidated by such a rich panoply of data (Baumeister, 2013). A narrative review lowers the risk of reproducing a single study's limitations and can broaden the potential conclusions (Baumeister and Leary, 1995). In this case, behavioral and physiological observations, as well as longitudinal and cross-sectional study designs aided in reducing potential biases stemming from a single design.

From a methodological perspective, the predominance of self-report measures from the articles offered rich feedback from the participants' internal states. However, this may represent a potential limitation of the present review. Such self-report measures could indeed introduce systematic shifts in the pattern of results stemming from issues such as the social desirability of curiosity. The anonymity of the questionnaires may also influence the pattern of results. Finally, the samples in the studies reviewed included a majority of young adult participants from the United States. Luckily, some studies had participants from Japan, China, the United Kingdom, and Germany as well as from older age groups. This built a more robust sample that permits a certain level of generalizability. However, the samples include a sizable proportion of university students, which is but a small demographic of the global population. Furthermore, studies conducted since 2020 were inevitably affected by the COVID-19 pandemic.

## 5 Conclusion

The literature reviewed above showed a clear pattern of results indicating the generative potential of IPC for relationships, health, and wellbeing. Engaging in frequent IPC behaviors can require great courage and vulnerability, the effects of which can be felt, appreciated, and learned by others. As many of the authors underscored, curiosity is neither always good nor always bad, but must be understood within its individual-situational-environmental context.

This review has identified the many ways in which IPC can lead to positive outcomes, while providing the reader with tips and tricks to develop IPC and its associated adaptive, exploratory behaviors. Adopting a more curious mindset could allow more growth, acceptance, communication, and learning in the face of the unknown, rather than resistance, defensive coping, and rejection. IPC coupled with true empathetic openness to another's experience allows narratives and stories to find their place and hold shared vulnerabilities. For all these reasons, modeling adaptive IPC may be one of the most precious gifts one could hope to offer humanity.

## Author contributions

MLJ: Conceptualization, Data curation, Formal analysis, Investigation, Methodology, Project administration, Resources, Visualization, Writing – original draft, Writing – review & editing. HL: Supervision, Writing – review & editing.

## Appendix A

**Table A1:** Evaluation criteria to identify final articles.

5	Interpersonal curiosity as a topic
4	Link to curiosity may provide useful support but no direct link to interpersonal curiosity or safe relational spaces
3	Not directly related to interpersonal curiosity but could be linked to research questions
2	Indirect link with interpersonal curiosity and relational spaces
1	No link with curiosity or interpersonal relational spaces
